# Penile Nodule: What's Your Diagnosis?

**DOI:** 10.7759/cureus.20270

**Published:** 2021-12-08

**Authors:** Giridhar Guntreddi, Jayasree Vasudevan Nair, Neelima P Theella, Swayam P Nirujogi

**Affiliations:** 1 Pediatrics, Sanford Health, Bemidji, USA; 2 Pediatrics, Sutter Health, Jackson, USA; 3 Family Medicine, Independent Researcher, Franklin, USA; 4 Family Medicine, Tower Health Medical Group, Reading Hospital, Reading, USA

**Keywords:** bimanual retractile manoeuvre, penile cysts, manual retraction, circumcision, smegma pearl

## Abstract

Smegma pearls can cause diagnostic dilemmas for pediatricians who are unfamiliar with this condition leading to unnecessary investigations and referrals. Despite the common occurrence of smegma pearls in uncircumcised young boys, it is not often reported in the literature. Smegma is a normal secretion consisting of desquamated epithelial cells, fat, and protein. It has mixed bacterial flora with smegma bacillus in 50% of cases. Smegma itself is neither damaging nor irritating substance and as Howe has stated, it is not carcinogenic also. Smegma production and keratinization of cells facilitate the separation of the fused foreskin from the glans epithelium. we are presenting a case of a penile nodule in the shaft of the penis without any pain, bleeding, or discharge. The smegma content gives a yellowish color to the lump. Smegma pearls do not have any covering sac. When the smegma is covered in a well-formed epithelial wall, it is called a smegma cyst. Long-standing smegma collection can turn into a hard stone-like structure called Smegmolith due to chronic irritation and mineral accumulation. Treatment should include monitoring for resolution with parental reassurance. Smegma pearls are benign, and they spontaneously resolve over time. This case report intends to help pediatricians correctly identify this benign, yet not widely published in the literature condition and reassure the parents and patients to improve the patient care and avoid unnecessary tests and referrals.

## Introduction

Smegma pearls [1] can cause diagnostic dilemmas for pediatricians who are unfamiliar with this condition leading to unnecessary investigations and referrals. Despite the common occurrence of smegma pearls in uncircumcised young boys, it is not often reported in the literature. Smegma is a normal secretion consisting of desquamated epithelial cells, fat, and protein [2]. It has mixed bacterial flora with smegma bacillus in 50% of cases. Smegma itself is neither a damaging nor irritating substance and as Howe [3] has stated, it is not carcinogenic also. Smegma production and keratinization of cells facilitate the separation of the fused foreskin from the glans epithelium. The preputial undersurface and the surface of the glans share a common epithelium during embryological development. So, the foreskin is fused to the glans in most children at birth. Over a period, the separation process will be complete by breaking down the foreskin adhesions. During this process, incomplete breakdowns of adhesions can lead to pockets where smegma can get entrapped. The entrapped smegma can transform into a nodule or lump called smegma pearl [4].

## Case presentation

**Figure 1:**
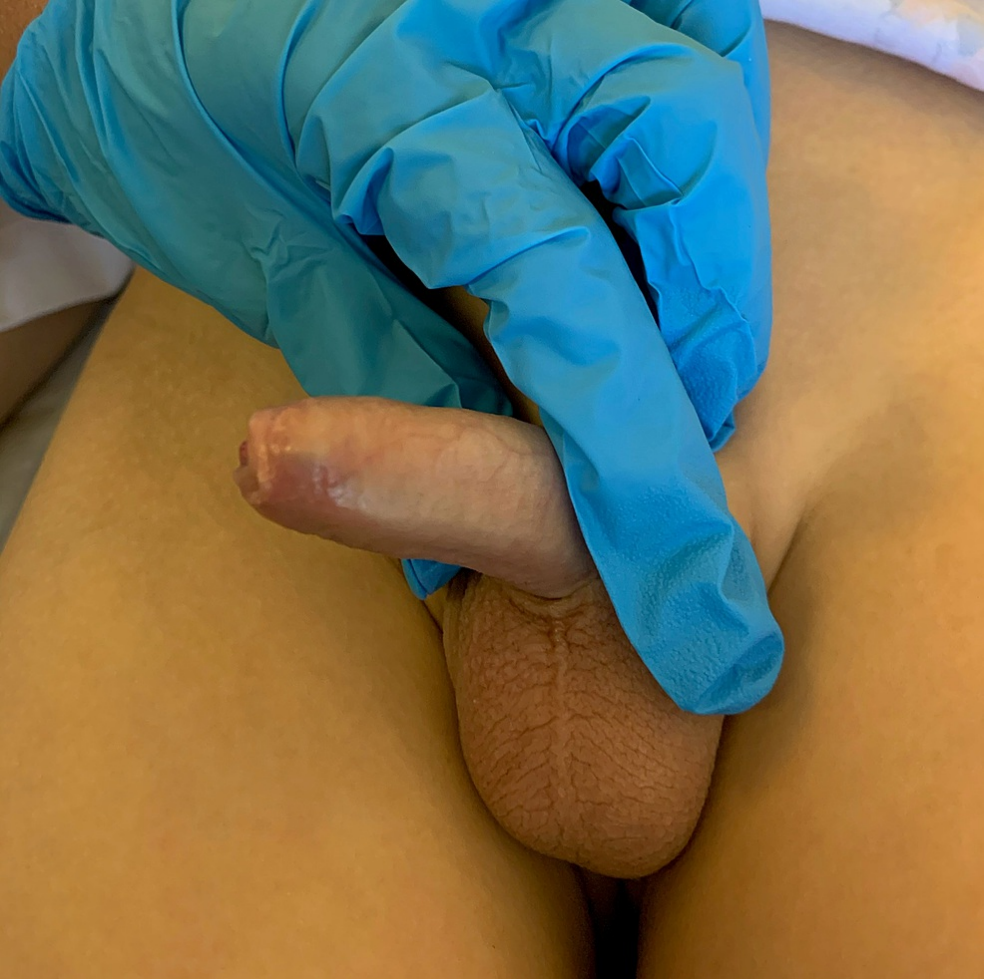
Penile lesion is resolved completely.

A healthy four-year-old boy was brought to the pediatric clinic by his apprehensive mother for concern of a nodule on his penis. The mother noticed this lesion one day before the presentation. The boy was asymptomatic without any pain or discomfort from the lesion, and he denied any urinary symptoms. The mother denied any trauma. He had no lesions on other parts of his body. His past history is noncontributory, as is his family history. His growth parameters and vital signs were normal. The boy's examination was normal other than the lump on his penis. The lump was noted under the foreskin on the left side. It was a 1x1 cm size round nodule with a yellow hue, oblong shape with its axis along the axis of the penis (Figure 1).

**Figure 2:**
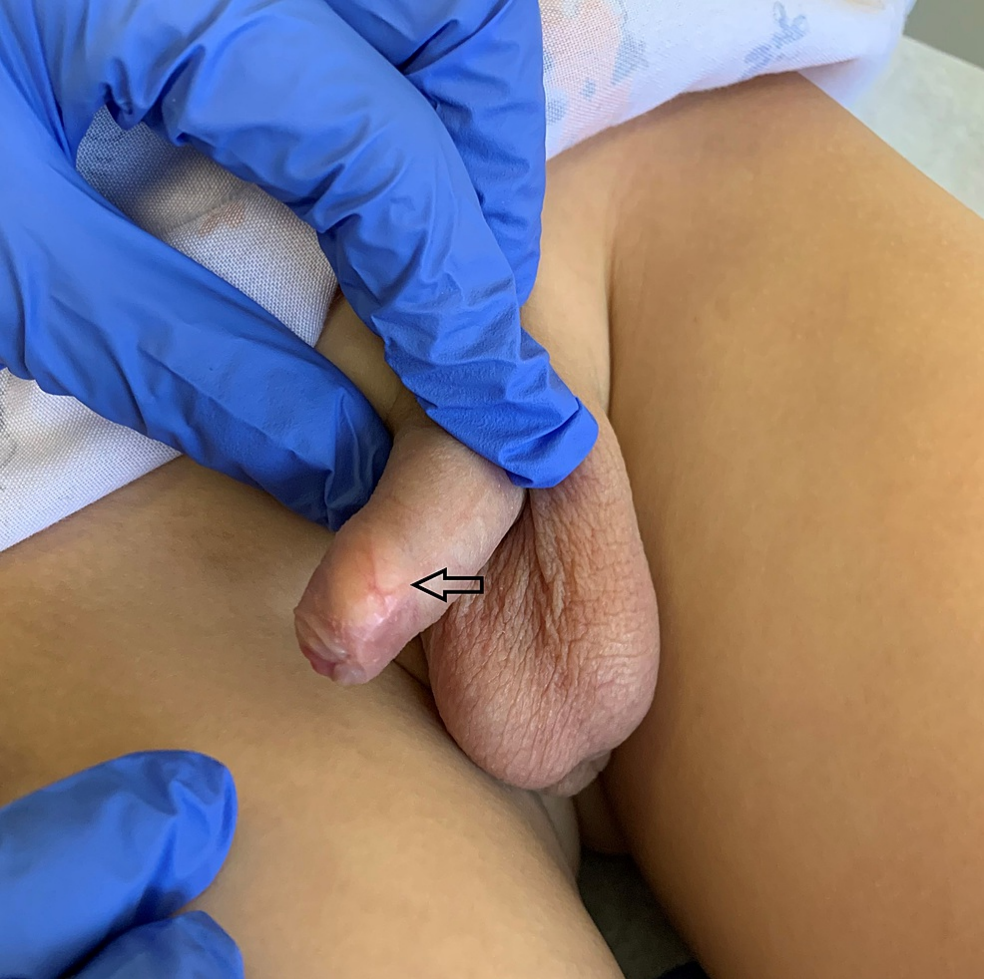
Lateral view of a mobile yellow nodule (black arrow) between the foreskin and the glans penis of the uncircumcised boy.

The lesion was nontender, soft in consistency, and was freely mobile. His foreskin was partially retractile. Based on the appearance and clinical picture, a diagnosis of smegma pearl was considered. An Ultrasound was done, which excluded the cystic or solid lesions and noted a soft tissue thickening at the area, further confirming the diagnosis of Smegma pearl. The benign etiology and spontaneous resolution were discussed with the mother, and she was reassured. we did not try the BRM (bimanual retractile manoeuvre) technique to assist the resolution by manual expression of smegma by gentle pressure, reported by Sonthalia and Singal [1], after discussing with the mother and pediatric urologist. After four months, when he came for follow-up, the lesion was resolved entirely (Figure 2).

## Discussion

However, there is no definite explanation for why it develops as a nodule in some boys. Smegma pearls are yellowish-white lumps noticed in uncircumcised boys, most commonly on the ventral surface of the glans. They are seen anywhere between the preputial membrane's attachment to the glans and the base of the glans penis. The urethral meatus is not affected, and the prepuce is partially retractable in all cases. The smegma content gives a yellowish color to the lump. Smegma pearls do not have any covering sac. When the smegma is covered in a well-formed epithelial wall, it is called a smegma cyst. Long-standing smegma collection can turn into a hard stone-like structure called Smegmolith due to chronic irritation and mineral accumulation. It is seen in older people. Treatment should include monitoring for resolution with parental reassurance. Bimanual retraction and manual expression of smegma is possible but poses a theoretical risk for paraphimosis, a urological emergency. This procedure can lead to possible irritation of the foreskin, recurrence of phimosis, and ultimately recurrence of smegma pearl (Table 1) [5].

**Table 1:** Differential diagnosis of penile cysts.

Acquired (False or Pseudocyst)	Congenital (True cyst)
Smegma cyst	Congenital Dermoid cyst
Inclusion dermoid cyst	Median raphe cyst
Prepucial Epstein pearls [6]	Para meatal cyst [7]
Trichilemmal cyst	Mucoid cyst [8]
	Epidermoid cyst
	Pilosebaceous cyst
	Juvenile Xantho granuloma

Preputial Epstein Pearls are the preputial counterpart of palatal Epstein pears in neonates. It is a benign condition due to the collection of keratinized epithelial cells during fetal development. The incidence of preputial pearls has been reported to be 7.3 per 1000 live-born male neonates among Indian children [6]. Prepucial cyst arises from prepucial skin and therefore it is also called as Keratin pearl. Median raphe cysts are thought to be due to embryologic developmental defects in the closure of the urethral folds. It is always located on the ventral side of the penis in the midline. Epidermoid cysts are formed from the trapped keratin produced by the surface epidermal cells and follicular cells, which are moved into deeper layers of the skin. Epidermal inclusion cysts are acquired cysts on the prepuce or its remnants secondary to penile surgeries, including circumcision. Pearly penile papules are seen as pearly papules around the coronal margin of the glans. This benign condition is seen in adult men. small Para meatal cyst is asymptomatic and does not require urgent interventions [7]. Surgical extractions of the entire sac were shown to be a reliable way of symptomatic management and had an excellent prognosis. Mucoid penile cysts are rare benign lesions. The cysts most commonly arise from ectopic urethral mucosa sequestered during embryologic development. The cysts are small, soft, and freely movable masses. They are asymptomatic unless they are complicated by infection or obstruction [8].

## Conclusions

Smegma pearls are benign, and they spontaneously resolve over time. Smegma is a naturally occurring accumulation of dead skin cells and oily secretions around the foreskin in males and clitoris in females. Smegma can be controlled easily by good personal hygiene and this should be encouraged to all. Bimanual retraction and manual expression of smegma is possible but pose a theoretical risk for paraphimosis. This procedure can lead to possible irritation of the foreskin, recurrence of phimosis, and ultimately recurrence of the smegma pearl. This case report intends to help pediatricians correctly identify this benign, yet not widely published in the literature condition and reassure the parents and patients to improve the patient care and avoid unnecessary tests and referrals. 
